# *“You do it to cover your own back”:* The assessment of cervical spine radiculopathy among physiotherapists in the United Kingdom: A mixed methods research study

**DOI:** 10.1371/journal.pone.0325922

**Published:** 2025-07-02

**Authors:** Michael Mansfield, Stephanie T. Jong, Toby Smith

**Affiliations:** 1 Faculty of Medicine and Health Sciences, University of East Anglia, Norwich, United Kingdom; 2 School of Sport, Exercise and Rehabilitation Sciences, College of Life and Environmental Sciences, University of Birmingham, Birmingham, United Kingdom; 3 Warwick Medical School, University of Warwick, Coventry, United Kingdom; University College Dublin - National University of Ireland: University College Dublin, IRELAND

## Abstract

Cervical spine radiculopathy [CSR] is a complex condition that is challenging to diagnose. The assessment methods used by United Kingdom [UK] physiotherapists to diagnose CSR remain unclear. A mixed-methods explanatory sequential design was used to investigate the assessment strategies that UK Health and Care Professions Council HCPC physiotherapists use and the reasons behind this decision-making in clinical practice. Phase 1 of the mixed methods research [MMR] study was a national online survey. The 63 respondents reported that the most common assessment strategies included muscle strength [94%], light touch sensation [78%] and reflex testing [89%]. Phase 2 of the MMR study included 11 qualitative interviews with Phase 1 [survey] participants. Four themes were established: perception of role, service constraints, minimising risk, and understanding symptoms. Physiotherapists often explained decision making in practice is based upon individual and organisational barriers. The choices available to physiotherapists may be based on cost, departmental knowledge and skill or convenience. However, the best available evidence suggests that physiotherapists should continue to use a biopsychosocial approach when establishing a CSR diagnosis. Physiotherapists should continue to embrace all assessment strategies available and strive to enhance or change practice.

## Background

Radiculopathy is a type of peripheral neuropathy that occurs when a spinal nerve or its root is compressed or when the blood supply or nutrition to a nerve axon or its root is interrupted [1–3]. The incidence rate of cervical spine radiculopathy [CSR] is reported as 0.8 to 1.8 per 1000 person-years and prevalence ranges from 1.2 to 5.8 per 1000 people [4]. People with CSR may describe sensations of burning, tingling, sensitivity to mechanical pressure [light touch, blunt and/or sharp stimuli], shooting or electric shock-like pain, thermal [hot, warm and cold] sensitivity, numbness or a combination of these [5–7]. The variation in clinical presentation also reflects the multiple etiologies and underlying mechanisms in peripheral neuropathies [8].

There is currently no agreed CSR diagnostic consensus. Physical tests used in the assessment of CSR may include tendon reflexes, manual muscle testing of key muscles, sensory testing, range of motion and symptom provocative tests or any combination of these [1,9–11]. However, the reliability of identifying CSR in this way is based on a small number of heterogeneous studies with mixed findings [10,12]. After examining five low-quality studies, a systematic review assessed the ability of physical examination tests to diagnose CSR [1]. Furthermore, there are inconsistencies in testing procedures and interpretation [12]. After examining five low-quality studies, a systematic review assessed the ability of physical examination tests to diagnose CSR (1). Among the three studies (n = 350) that evaluated the Spurling test (which typically involves cervical extension, lateral flexion toward the symptomatic side, and the application of downward axial compression), the movements performed prior to axial compression varied slightly, for example, some studies included cervical rotation in addition to extension and lateral flexion. In contrast, others differed in the order or extent of movement. The review reported moderate sensitivity and high specificity [Sensitivity [Se] 0.65, 95% CI: 0.49–0.79; Specificity [Sp] 1.00, 95% CI: 0.56–1.00] and [Se 0.38, 95% CI: 0.22–0.56; Sp 0.94, 95% CI: 0.83–0.99] respectively [1]. Screening tools such as Neuropathic Pain Questionnaire [NPQ] [13], Identification [ID] Pain [14], Leeds Assessment of Neuropathic Symptoms and Signs [LANSS] [15], Douleur Neuropathique 4 [DN-4] [13] or PainDETECT [16] can identify underlying pain mechanisms in radiculopathy presentations. Investigating the utilisation of these screening tools or questionnaires in physiotherapy practice has not been completed to date. The clinical expression of sensory and motor symptoms of CSR may not always conform to standard ‘textbook’ descriptions. To assess somatosensory integrity and function, both large and small sensory nerve fibres should be evaluated since both may be impaired in radiculopathy presentations [Schmid et al., 2020]. It is not only the causes that differ but also the clinical expression of CSR symptoms. This can be achieved through somatosensory bedside examination, patient-reported outcomes, and quantitative sensory testing. However, it remains unclear which somatosensory testing methods are commonly used in clinical practice, and by which clinicians, to support diagnostic formulation and identify underlying pain mechanisms. Furthermore, it remains unclear if CSR presentations always exhibit predominantly neuropathic pain [NeP] components, as they can be classified as mixed-pain syndromes but can also be categorised as a NeP condition. Exploring the use of diagnostic methods in clinical practice and the reasons underpinning these decisions are therefore investigated in this mixed methods study.

The explanatory sequential design was selected for this MMR phase of research. An explanatory sequential design requires collecting and analysing quantitative data to identify areas that require further explanation through qualitative research. It usually involves a purposive sampling approach to select participants [17,18]. Explanatory sequential design is appropriate for researchers who are more focused on quantitative methods and who have already identified a variable to measure [18]. Providing an explanation behind the reported decisions made in the quantitative survey was appropriate to explain through qualitative interviews. Owing to the consideration of the complexity that existed in the professional working environments, decisions on CSR assessment practices are sets of belief systems, attitudes and healthcare systems that have, in some way, impacted participants in this study. It enabled the comparison and communication of congruent and divergent interpretations and facilitated the drawing of conclusions. This mixed methods research promoted reflection on how quantitative and qualitative methods are integrated. Integration occurred at the interpretation and reporting level through joint display. At the interpretation and reporting level, integration refers to combining two data sets to provide a more comprehensive understanding than either data set alone [18]. This type of integration can take various forms, such as presenting both quantitative and qualitative data in a report, converting one type of data to another [for example, transforming qualitative data into quantifiable data] and integrating it with non-transformed data [for example, combining quantified qualitative data with existing quantitative data].

This mixed methods study aimed to examine the assessment approaches employed by UK physiotherapists in clinical practice and explore the rationale behind their decision-making when establishing a CSR diagnosis. The first phase [online survey] aimed to answer: (i) What are the current physical assessment strategies (including questionnaires and screening tools) used in UK physiotherapy practice to inform the diagnosis of CSR and (ii) what are the barriers and facilitators in using clinical assessment strategies in practice? The second phase aimed to explain the survey findings through qualitative interviews.

## Methods

### Study design

This study followed a mixed methods research approach with an explanatory sequential design [17]. It was conducted in two phases, Phase 1: a quantitative cross-sectional survey and Phase 2: qualitative semi-structured interviews. Phase 2 was conducted to help explain and elaborate the results from Phase 1, refining the results and exploring UK physiotherapists’ views and experiences in greater depth. Integration between the Phase 1 and 2 results occurred in two steps. The initial step involved utilising the survey findings to guide the qualitative interviews. The second step combined both sets of related results and derived cohesive conclusions. We adhered to the Good Reporting of A Mixed Methods [GRAMM] study checklist [18] for transparency related to data collection methods, sequencing, sampling, points of integration, and data analysis techniques.

### Phase 1 – Survey study

#### Recruitment.

A snowball sampling approach was adopted. From the research team’s professional accounts, the survey was advertised through X [formerly Twitter], Professional special interest group webpages [including the Interactive Chartered Society of Physiotherapy – iCSP, Association of Trauma and Orthopaedic Chartered Physiotherapists [ATOCP], Musculoskeletal Association of Chartered Physiotherapists [MACP], Physiotherapy Research Society [PRS] shared at least one advertisement to recruit prospective participants. The survey was open from 30/01/2022-01/03/2022.

Eligibility: Health and Care Professions Council [HCPC] [19] UK Physiotherapists working in musculoskeletal and/or orthopaedic clinical settings.

Sample size: The number of HCPC registered physiotherapists in 2021 was 61,760, though not all of these physiotherapists will have experience or work in clinical settings assessing people with CSR. At the time of writing this study, there was no data available on what percentage of these physiotherapists assess and/or manage adults with CSR. The Musculoskeletal Association of Chartered Physiotherapists [MACP] is a clinical interest group [CIG] and professional network of physiotherapists who have worked or have worked in musculoskeletal physiotherapy practice-based settings. Its membership is 1,200 physiotherapists. The majority are based in the UK. However, it is acknowledged that physiotherapists may choose not to become members of this CIG but will have experience in the assessment and management of CSR. Therefore, an estimated population size of 2,000 respondents was set. The sample size calculation was made using the ‘Sample Size Calculator platform [https://www.qualtrics.com/blog/calculating-sample-size/], considering a 95% confidence level and a margin of error of 5%. The response target was 323 respondents. The survey was open for four weeks [January 2022-February 2022].

### Survey development and data collection

The survey was designed to determine the usage and possible barriers and facilitators of common assessment procedures for CSR (S1 File). The survey was piloted with two physiotherapists. The piloting feedback facilitated appropriate amendments to question formatting and technical language with some somatosensory tests.

### Data analysis

The data were imported into Microsoft Excel and data analysis was performed using SPSS statistical software [IBM Corp, Chicago, Illinois, USA]. Descriptive statistics were used to analyse the data, including mean, median and variances for the sample. Free-text comments or answers were further explored in qualitative interviews.

### Phase 2 – Qualitative interview study

#### Recruitment.

Eligible participants were registered HCPC UK Physiotherapists who completed the Phase 1 survey and responded ‘yes’ to being contacted to complete a qualitative interview. The lead researcher [MM] invited each prospective participant one-by-one to take part in an interview. The qualitative interviews were completed between 01/10/2022–30/12/2022.

When conducting qualitative research, it is important to ensure that the sample’s composition and size are appropriate and adequate [20]. This is known as sample adequacy, and it plays a crucial role in assessing the reliability and credibility of the research [21,22]. However, determining the ideal sample size for qualitative research is a topic of debate [23]. Unlike quantitative research, which relies on statistics-based rules, the complexity of qualitative research stems from the various methodological, theoretical, epistemological, and ideological perspectives involved [23]. It is acknowledged that sample size alone is not the only factor when undertaking qualitative interviews [20]. The concept of ‘saturation’ is debated when determining sample size for qualitative interviews [24] and guidance on assessing saturation and the sample sizes needed to reach saturation have been vague. Vasileiou et al. [2018] [24] conducted a systematic review of qualitative studies using in-depth interviews in health-related journals over a 15-year period and found the vast majority of articles did not justify their sample size. Where justifications were given, saturation was cited in 55% of articles; however, claims of saturation were not substantiated in relation to study procedures [24]. Therefore, the lead author [MM] reflected and reviewed the data quality after each completed interview and employed a lower limit of eight participants and an upper limit of 16 participants [25]. This informed iterative refinement of the topic guide and supported the decision to employ a lower limit of eight participants and an upper limit of 16 participants [25], aiming to generate a rich, complex narrative for analysis [26].

### Qualitative interview development and data collection

The lead researcher [MM] conducted all 11 individual semi-structured interviews, each interview lasted between 45–60minutes. The topic guide was informed by the survey responses and relevant systematic reviews [4,10,11] (S2 file). The topic guide was piloted with a physiotherapist based in UK clinical practice who was not recruited in the qualitative study. The pilot interview provided feedback on the question order, answers, follow-up questions, syntax and flow of the interview. Minor iterations of the topic guide occurred throughout subsequent interviews and research team meeting discussions [e.g., a focus on exploring survey findings and less focus on the interviewee’s personal clinical practices].

### Lead researcher positioning

The lead researcher [MM] is a Health and Care Professions Council [20] registered physiotherapist with 17 years of clinical academic experience, employed as an academic at the University of Birmingham and a PhD student at the University of East Anglia. Therefore, it is acknowledged that interview participants may perceive an implicit or explicit power balance. The lead researcher [MM] completed a reflective journal and decision-making audit trail monitoring possible impact with conscious or subconscious bias, belief systems and personal professional experience. These documents were discussed as part of an ongoing peer-review process with other research team members [TS, SJ]. The lead researcher [MM] received training from an experienced qualitative researcher [SJ] and the university’s post-graduate qualitative training programme.

### Data analysis

A six-phase reflexive thematic analysis was used [22]. Interviews were audio and video recorded, transcribed verbatim and anonymised by the lead researcher [MM]. To ensure comprehensive familiarity with the data, the lead researcher [MM] read each transcript multiple times and listened to the audio files while reading. This allowed the lead researcher [MM] to make notes on any important information [such as any overlaps in speech, interfering noise and differing styles of speech, for example, distinguishing between “I don’t, no” and “I don’t know”]. An online reflective journal was used to record and reflect on any insights or new ideas that arose while becoming familiar with the material.

In the next phase, coding occurred in two stages – initial and focused. In the initial coding of transcripts, the lead researcher [MM] reviewed each transcribed line carefully and inductively coded topics such as rationale, reasoning, motivations or areas that addressed the study’s aim and objectives. Initially, the data were coded manually by highlighting, colour-coding and labelling certain sentences to help interpretation of the data. A list of these codes was compiled before writing a brief description of each code to ensure to remember its meaning.

In focused coding, the lead researcher [MM] pursued a selected set of central codes throughout the entire dataset and the study [27]. This required making decisions about which initial codes were most prevalent or important, and which contributed most to research questions being answered [27]. A project coding list and codebook [group of coding themes] was developed to interpret any given text accurately.

Two researchers [MM, SJ] coded three interviews independently for investigator triangulation [28]. The researchers [MM, SJ] met to discuss the codes. This approach enhances consistency in coding, and clarifies interpretations, and inferences [29]. Detailed information on the analysis process was recorded in an audit document. The codes and overall themes were then discussed and negotiated among MM and SJ in a final discussion to reach a consensus. During the analysis, the lead researcher [MM] made fieldnotes of reflections and interpretations. To enhance the credibility of the methods used, this study was guided by a 15-point checklist of criteria for conducting good thematic analysis [29] detailed in S3 File.

### Mixed methods research integration process. Survey and interviews

For this mixed methods research, integration occurred at the interpretation and reporting level through joint display. Joint display is a visual representation that a researcher can use to present both quantitative and qualitative data analyses or results in a single display [30,31]. Creating an effective joint display in the analysis stage involves selecting specific qualitative and quantitative data to use, choosing the appropriate format for each type of data, adjusting the organisation of the two types of data, and using creative formatting techniques to obtain a deeper understanding and meaning of the mixed data findings.

## Results

**Fig 1:**
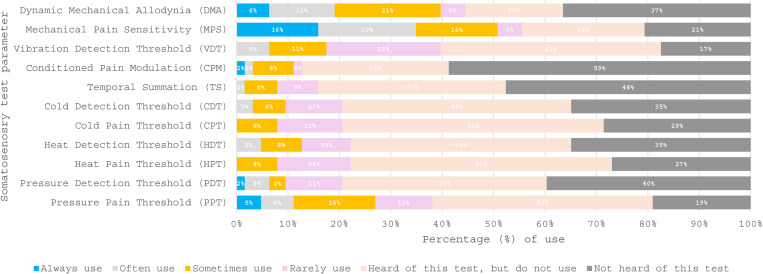
A stacked bar chart to illustrate the frequency use across all somatosensory test parameters when establishing a cervical spine radiculopathy diagnosis (n = 63).

**Fig 2:**
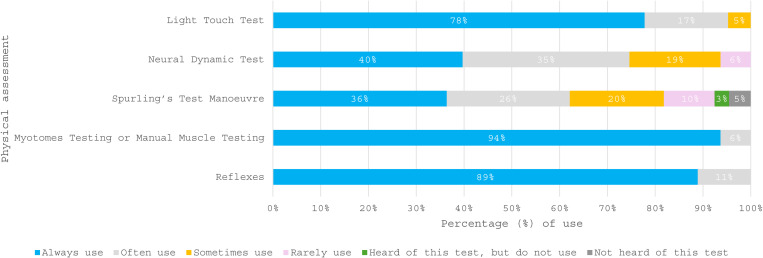
A stacked bar chart to illustrate the frequency of use across physical assessment testing methods when establishing a cervical spine radiculopathy diagnosis (n = 63).

Sixty-three completed surveys were analysed. Table 1 details the healthcare sector, clinical specialty, highest education award and length of time working as a physiotherapist across the respondents. From the 63 survey respondents, 75% [47/63] self-reported working in the NHS. Twenty-eight [44%] worked in primary care and nineteen [30%] worked predominantly in secondary care. Eleven of the 63 respondents [18%] worked in private practice and one respondent worked in a military healthcare setting. Fifty-six [89%] reported that their clinical speciality was musculoskeletal. Three [5%] and two [3%] of respondents specialised in musculoskeletal orthopaedic and pain management, respectively. Sixteen of the 63 survey respondents agreed to be contacted about the qualitative interviews. A total of 16 physiotherapists expressed an interest in being contacted; all were individually contacted by email. A total of 11 physiotherapists were recruited over two months for the qualitative interview phase. The remaining five survey respondents did not respond to further interview requests. The participant characteristics are reported in Table 2. Figs 1,2 present which assessment strategies are used when establishing CSR diagnosis. The mixed methods synthesis identified four main qualitative themes [perception of physiotherapist role; service constraints; minimising risk to the patient, clinician and service; understanding symptoms], which demonstrate the connections between physiotherapists’ experiences, internal and external factors, and their diagnosis strategies when establishing CSR in clinical practice. The themes that contributed to each qualitative theme are listed in S4 File.

**Table 1:** Survey Participant Demographics.

Healthcare sector	Number [n]	Percentage [%]
National Health Service - Primary care	28/63	44.4
National Health Service – Secondary care	19/63	30.2
Private practice	11/63	17.5
Military	1/63	1.6
Other	4/63	6.3
Clinical specialty		
Clinical – Musculoskeletal	56/63	88.9
Clinical – Orthopaedics	3/63	4.8
Clinical – Pain management	2/63	3.2
None of the above	2/63	3.2
Highest education award		
BSc	16/63	25.4
MSc [Pre-registration]	10/63	15.9
MSc [Post-registration]	16/63	25.4
Post graduate certificate	8/63	12.7
Post graduate diploma	6/63	9.5
Graduate diploma in physiotherapy	4/63	6.3
MRes	1/63	1.6
PhD	1/63	1.6
Professional doctorate	1/63	1.6
Length of time working as a physiotherapist		
Less than one year	2/63	3.2
1 to 5 years	6/63	9.5
6 to 10 years	7/63	11.1
11 to 15 years	19/63	30.2
16 to 20 years	11/63	17.5
More than 21 years	18/63	28.6

**Table 2:** Qualitative interview participant demographics.

Participant number	Healthcare sector	Highest education award	Length of time working as a physiotherapist
001	National Health Service – Primary care	MSc	6 to 10 years
002	Private practice	MSc	More than 21 years
003	National Health Service – Primary care	BSc	1 to 5 years
004	National Health Service – Secondary care	MSc	6 to 10 years
005	National Health Service– Secondary care	PhD	16 to 20 years
006	National Health Service – Primary care	MSc	6 to 10 years
007	National Health Service – Primary care	MSc	More than 21 years
008	Private practice	BSc	More than 21 years
009	National Health Service – Primary care	MSc	16 to 20 years
010	National Health Service – Primary care	MSc	16 to 20 years
011	National Health Service – Secondary care	MSc	16 to 20 years

### Theme 1: Perception of physiotherapist role

Consistent from survey and interview data, participants made decisions based on four categories: CSR assessment informed by research, continuing professional development training needs and their clinical experiences. Manual muscle testing was identified as an important assessment method, reported as always or often used by all survey respondents. When explored further in the interview data, all interview participants justified their use of muscle strength testing, light touch sensation and reflexes in clinical practice as these techniques were considered easy to use and not time-consuming given their short appointments. This is illustrated by one participant:

*“…we operate on 30-minute appointments, every minute counts when assessing a patient with CSR. I am not surprised that some of these somatosensory tests are not used, they are too long to complete in practice”*

*[Participant 003.Qualified for 1-5 years and working in primary care]*

Most interview participants reported that their service required robust data to change individual or departmental assessment practices before adopting more logistically challenging tests such as small fibre testing. One interview participant, a physiotherapy service lead, explained that they were not surprised by the survey findings as they were unaware of published literature that supported the use of small fibre somatosensory testing when compared to muscle strength testing, light touch sensation and reflex testing:

*“I would be surprised if physiotherapists used any of these [somatosensory] tests in practice… I am not aware of any data that would mean we should change our assessment tests from what we are doing already…. ”*

*[Participant 007.Qualified for more than 21 years and working in primary care]*

Of 63 respondents, 40 [64%] prioritised other assessment measures instead of pain questionnaires or tools when making a CSR diagnosis. Sixteen respondents [26%] believed that the questionnaires or tools were more useful for researchers than for clinicians, while 30% lacked confidence in using and interpreting the collected data [17/63]. Fifty-one survey respondents [81%] reported that providing online access to free pain screening tools or questionnaires could increase their usage in clinical practice. Moreover, forty-two [67%] and thirty [48%] survey respondents stated that additional training on the appropriate use and timing of these tools or questionnaires might further enhance their utilisation.

All interview participants supported the survey results that if expectations of pain questionnaires or other somatosensory testing are to be introduced into practice, formal and informal continuing professional development education would be necessary. This is exhibited by the following participant:

*“Pain education is poor in physio education; I don’t remember any education being provided around different sensory tests or screening tools at university… I was aware of these when I qualified and had a mentor to show me”*

*[Participant 002.Qualified for more than 21 years and working in private practice]*

All interview participants explained that the clinical assessment of patients is more valuable to physiotherapists than the outcomes of questionnaires or small-fibre somatosensory testing data. This was influenced how other multi-disciplinary team clinicians assess CSR and pattern recognition reasoning:

*“I learnt about neuro assessment by observing an ortho surgeon, they don’t use any questionnaires or heat tests…. I’ve adopted this in my triage assessments because I make referrals to ortho [orthopaedics]”*

*[Participant 004.Qualified for 6-10 years and working in secondary care]*

Another physiotherapist shared this sentiment.

*“I think that as you get more mileage as a spinal physiotherapist, you see patterns of presentations… the subjective assessment tells you what you want to know, and the physical assessment is for the patient’s reassurance really”*

*[Participant 006.Qualified for 6-10 and working in primary care]*

Most interview participants explained that physiotherapists may prioritise other assessment strategies because of the expectations of their line managers or the department they work in. This is exemplified with this participant:

*“There is a difference across different departments, it depends on what the priorities of the manager are. Working in one department with a research focus meant we collected a lot of questionnaire data which helped researchers”*

*[Participant 008.Qualified for more than 21 years and working in private practice]*

Whereas some interview participants explained how working as part of a multi-disciplinary team and the clinical lead’s expectations influenced decision-making when assessing CSR. Noteworthy, all five reported that orthopaedic surgeons were clinical service leads:

*“My orthopaedic surgeon I work with would expect me to complete a basic neuro of myotomes, dermatomes and reflexes for every patient… this is foundation level triage assessment”*

*[Participant 003.Qualified for 1-5 years and working in primary care]*

### Theme 2: Service constraints. Healthcare system barriers to CSR clinical assessment

Survey respondents reported how time availability with patient appointments, the integration of testing and questionnaires into IT systems, the differences between public and private sector healthcare and the cost and availability of equipment, influence the decision-making and justification of assessment strategies for CSR. These factors were presented as response options within the survey, facilitating participants to indicate their relevance to their decision-making processes. Time availability in clinical practice to collect data from small fibre somatosensory testing [38/63; 60%] or pain questionnaires or tools [38/63; 60%] was reported as a reason why these are not considered when forming CSR diagnoses. Thirty-nine [70%] survey respondents reported that having more time in clinical practice would enhance the use of screening tools or questionnaires. Whereas, 56% [35/63] indicated that increased assessment time in clinical settings could improve the use of small fibre somatosensory testing in practice. Interview participants suggested that these findings explained the selection of assessment strategies:

*“I am not surprised the survey reported this; muscle strength, dermatomes and reflexes are so quick to complete… we are under pressure with patient activity…The questionnaires would eat into assessment time…”*

*[Participant 010.Qualified for 16-20 years and working in primary care]*

Most interview participants, who worked in primary or secondary care, reported challenging CSR patient presentations. The interview participants attributed the complexity to co-morbidities [for example, anxiety symptoms], which made it challenging to prioritise assessment strategies and interpret findings, subsequently taking longer to reach a diagnosis. This is reflected by Participant 5:

*“The longer a person has symptoms, the more complex, this takes time as all aspects of their wellbeing should be considered…”*

*[Participant 005.Qualified for 16-20 years and working in secondary care]*

Despite this explanation, some interviewees explained that even if more time was allocated, they were unsure whether they would integrate further physical testing as they were focused on ensuring that patients received timely and accurate management pathways. Participant 9 explains this:

*“Even with more time, would the questionnaires change onward referral?…probably not…Reaching the right management pathway is most important when I see this patient…”*

*[Participant 009.Qualified for 16-20 years and working in primary care]*

Fifty-one survey respondents reported that providing online access to screening tools or questionnaires could increase their usage in clinical practice. When explored further in the qualitative interview, nearly all participants explained that the integration of questionnaires in clinical practice is mixed, and not all departments or healthcare information technology systems can embed them into practice:

*“The thing is, our IT systems are poor, the software to build questionnaires needs to be in place. We don’t need more pieces of paper, if it was IT-based I bet physiotherapists would use them more often”*

*[Participant 001.Qualified for 6-10 years and working in primary care]*

Some interview participants expanded on this, reporting that the timing of when to introduce questionnaires to a patient is unclear. They perceived that this lack of clarity negatively impacts clinicians and patients:

*“When would we ask the patient to complete these questionnaires…. Some patients don’t have IT access… and if [completed] in the waiting area, it would mean more time wasted”*

*[Participant 011.Qualified for 16-20 years and working in secondary care]*

Most interview participants had worked in public and independent healthcare sectors in the UK, where there were perceived differences in expectations of assessment and management strategies for CSR. The differences centered around access to specialised testing and the time it takes to order and complete assessments, such as imaging or nerve conduction studies:

*“There is more immediate access to assessment, like MRI the next day, that is why they may be used in practice more often”*

*[Participant 008.Qualified for more than 21 years and working in private practice]*

Some interview participants explained that some clinical departments have a research culture, which enhanced the uptake of screening tools and outcome measures. Participants reported that screening tools and outcome measures were selected due to existing service improvement projects or funded research studies that their department prioritised:

*“Yeah, I can imagine in some big departments there are multiple research studies that would collect the data, this can provide some ideas on the types of pain patients coming through the door…”*

*[Participant 001.Qualified for 6-10 years and working in primary care]*

One interview participant further explained, detailing how data collection using pain screening tools or questionnaires can support service managers in building a business case to justify physiotherapy services to commissioning groups.

All interviewees agreed with the survey findings that the low uptake of some somatosensory assessments related to the cost of equipment. Interview participants detailed equipment shortages in their departments, and small budgets to replace equipment. Participants perceived that decisions were based on availability of assessment equipment rather than clinical reasoning:

*“I currently carry my own reflex hammer, paid for by me with my name on it, that tells you why physios don’t decide to use anything else when assessing…”*

*[Participant 006.Qualified for 6-10 years and working in primary care]*

### Theme 3: CSR clinical assessment to minimise risk to the patient, clinician and service

Interview participants agreed with the survey results reporting that manual muscle testing, light touch sensation and reflex testing was always used in practice. When explored further, some interview participants explained that the reasons for this may be related to regulatory expectations and the influence of medico-legal claims:

*“As a bare minimum physio’s should complete muscle testing, reflexes and light touch sensation… it is what we are trained to do and if something went wrong, we can fall back on this – you do it to cover your own back”*

*[Participant 010.Qualified for 16-20 years and working in primary care]*

Participants explained that they would expect manual muscle testing, light touch sensation and reflex testing to be completed to meet clinical audit standards:

*“Our assessments get audited... the manager would want clear explanations as to why these haven’t been completed [if they haven’t]… it is not worth not completing these”*

*[Participant 003.Qualified for 1-5 years and working in primary care]*

### Theme 4: Physiotherapist understanding of CSR symptoms

Survey respondents reported how frequently pain detection or pain threshold testing are used when formulating a CSR diagnosis. Interview participants explained that CSR assessment focuses on two categories, pain symptoms and nerve descriptors. Interview participants reported mixed explanations of pain symptoms and nerve descriptors. A few participants rationalised that although pain is a common symptom with CSR, the physical testing often undertaken does not always relate to these symptoms. This was further detailed by some participants who described their role in understanding whether symptoms related to CSR were a result of a serious spinal pathology:

*“Pain is usually the main complaint with radiculopathy, but tests to assess pain are unreliable… I think it is more useful to understand whether its mechanical pain or non-mechanical pain… this helps to justify onward referral to surgeons or imaging for something nasty”*

*[Participant 010.Qualified for 16-20 years and working in primary care]*

Most interview participants stated that pain provocation tests are challenging to interpret because the symptoms may be persistent [lasting longer than three months], meaning that pain provocation tests have limited use when establishing a diagnosis, which was a finding from survey results:

*“….most patients coming through the service will have pain lasting more than three months… this means chronicity….that questions whether pain tests like Spurling’s is actually useful as they are already in pain before we start”*

*[Participant 004.Qualified for 6-10 years and working in secondary care]*

Understanding underlying pain mechanisms with CSR clinical presentations was important for a few interview participants. Despite this, the same interview participants reported that a possible explanation for why assessment strategies did not focus on pain symptoms may be attributed to physiotherapists’ perceived lack of knowledge related to underlying pain mechanisms.

“*These nerve tests should probably be used but I reckon most physio’s understanding of pain mechanisms is not good that why they are not used…”*

*[Participant 006.Qualified for 6-10 and working in primary care]*

All interview participants recognised that sensory changes, such as paraesthesia or dysesthesia, were likely to be descriptors reported by patients who had CSR. This was often asked in the subjective questioning stage of an assessment. There were, however, some differences in the explanation of how sensory symptoms related to nerve dysfunction should be assessed. Some interview participants explained that small fibre somatosensory testing would not change the treatment pathway for the patient with suspected CSR. Testing used by other healthcare professionals was explained as an important consideration when establishing CSR diagnosis. Several interview participants expanded on the reasons why some assessment practices, such as small fibre somatosensory testing and nerve-related pain assessment questionnaires, may depend on whether other clinicians in the management pathway utilise them:

*“My consultant does not use these tests… so we wouldn’t speak the same language if they were used”*

*[Participant 011.Qualified for 16-20 years and working in secondary care]*

## Discussion

This is the first mixed methods research study that has reported the assessment practices of UK HCPC physiotherapists when establishing a CSR diagnosis. Phase 1 survey results from 63 respondents identified that the most frequent assessment strategies used to diagnose CSR included manual muscle strength, light touch dermatomes sensation and reflex testing. The least frequent assessment strategies included thermal detection and pain thresholds, pressure detection and temporal summation somatosensory testing parameters. Survey participants reported that pain screening questionnaires or tools were rarely used in clinical practice to establish CSR diagnosis. Eleven qualitative interviews were completed in Phase 2 of the research program, and four qualitative themes contextualised the reasons assessment strategies were used to establish CSR diagnosis. The themes were perception of role, service constraints, minimising risk, and understanding symptoms.

Interview participants in this mixed methods research study explained that the reasons for selecting assessment strategies were related to pattern recognition reasoning strategies developed over years of clinical experience. Participants reported that large-fibre somatosensory testing was selected over small-fibre somatosensory testing because of previous CSR presentations assessed and managed throughout their clinical careers. Previous research found that those with more clinical experience and expertise tend to use inductive reasoning, while students and novice professionals rely on deductive reasoning [32]. Deductive reasoning is subject to a range of cognitive biases, including confirmation and availability bias [32,33]. This approach is often used by experienced professionals where patient presentations are straightforward. For students or novice physiotherapists, this reasoning approach increases the risks of inaccuracies and biases [34]. However, with an atypical clinical presentation, the expert, like the novice, will rely more on deductive clinical reasoning patterns [34]. Therefore, the results of this mixed methods research study can be interpreted that the interview participants rely upon pattern recognition reasoning for CSR diagnosis formulation. Notwithstanding this, it is acknowledged that clinical reasoning in assessing CSR cannot be separated from diagnostic decision-making and is thus a critical consideration for both recognising and managing CSR [35]. Despite the absence of robust evidence supporting its diagnostic validity, most HCPC-registered UK physiotherapists continue to primarily use neurological bedside testing to assess motor and/or sensory dysfunction when diagnosing CSR rather than relying on provocative testing. To date, only two studies Wainner et al. [2003] and Sleijser-Koehorst et al. [2020] have investigated this approach, both of which present methodological limitations. There is a clear indication for future research to investigate the understanding of novice and experienced physiotherapists’ clinical reasoning frameworks when assessing and managing people with CSR.

Survey respondents in this MMR study reported that small fibre somatosensory testing was not being used due to the time required for assessment, a lack of availability of equipment, and difficulties integrating IT systems for assessment results. During qualitative interviews, participants explained that they often faced time pressures due to the appointment time slots being 20–30 minutes long. Participants explained that they were likely to opt for assessment testing that was time efficient. This reasoning was also communicated in another study where physiotherapists were interviewed to determine the necessary skills, knowledge, and attributes for first-contact physiotherapists [FCP] in primary care [35]. Langridge [2019] [35] reported that physiotherapists who worked as FCPs had shorter appointment times than in their regular musculoskeletal physiotherapy roles. As a result, one of the key challenges these physiotherapists faced was making timely assessment decisions [35]. The time pressures for physiotherapists working in primary care can be attributed to the expectation that physiotherapists are required to evaluate multiple physiological systems, including musculoskeletal, neurological, cardiovascular and respiratory systems, to ensure safe care practices are followed [36]. All primary care and private physiotherapists that were interviewed were working as ‘first contact practitioners.’ As such, they have a responsibility of diagnosing whether a patient had CSR or non-specific neck pain with somatic referred pain. In contrast, if physiotherapists only saw patients referred by medical specialists who had already confirmed a CSR diagnosis, their diagnostic approach would differ accordingly. Despite this, it is important to note that somatosensory nervous system assessments should encompass both small and large fibre assessments to robustly assess nervous system integrity and function [37]. Pain perception is unique, yet neuropathic, nociceptive or mixed pain mechanisms can be features of CSR dependent on the pathophysiological mechanisms at play [37]. CSR can present as a painful or painless radiculopathy [37]. The phenotypic somatosensory sub-groups of people with CSR may account for some of the heterogeneity of presentation. People with CSR can have mixed, bi-directional sensory abnormalities, I.e., signs of a loss of function and a gain of function. People with CSR may experience sensory changes in the area where they feel the most pain [maximal pain area]. This includes a decrease in the ability to detect temperature, pressure, and vibrations, which explains why encompassing both small and large fibre assessments to robustly assess nervous system integrity and function. Selecting assessments based solely on time efficiency may mean that some CSR presentations are not assessed thoroughly and may result in an underdeveloped clinical diagnosis.

Within the current MMR study, survey respondents reported that assessment strategies selected to diagnose CSR were partly attributed to equipment availability and cost. This was further explained by interview participants, who explained that minimal equipment was required to complete large nerve fibre testing such as manual muscle strength, light touch dermatomes sensation and reflex testing [such as a reflex hammer and tissue paper]. Conversely, small fibre somatosensory testing, such as thermal detection and threshold, required specialised equipment unavailable in clinical departments. Quantitative sensory testing using the German Neuropathic Pain [DFNS] protocol requires specialised equipment, such as a Medoc machine [38]. However, these machines are expensive and require specialised training to operate and interpret [39]. Despite this, clinical bedside somatosensory testing using cotton wisp, tuning fork, metal, brush and von Frey filament is reported to be inexpensive [40]. Future research should consider validating bedside somatosensory assessment before implementing such an approach in clinical trials [40].

Interview participants in this study explained that medico-legal expectations related to CSR assessment decisions were, in part, related to guidelines or protocols from their local working environment, regionally or nationally. Some interview participants explained that not completing large fibre somatosensory testing may mean their assessment practice is vulnerable to exposure to medico-legal complaints. A physiotherapist’s decision to complete assessment tests or procedures based only on potential negative medico-legal implications risks stifling clinical reasoning [41]. Therefore, the justification to complete assessment testing for CSR diagnosis based on actual or potential medio-legal expectations requires careful reflection and well-reasoned justification. As such they have a responsibility to diagnose and identify patients with CSR.

The overuse of diagnostic tests by physiotherapists in primary care FCP roles has been attributed to the fear of missing serious pathology and concerns over litigation [42,43]. However, it is important to acknowledge that the potential for litigation resulting from missed diagnoses or delayed investigations is a valid concern. To avoid missing any serious spinal pathology, physiotherapists surveyed and interviewed in this study reported that using large fibre somatosensory testing or imaging to rule out any possibility of such pathology. This suggests that CSR may be considered a diagnosis of exclusion, which can create uncertainty among physiotherapists regarding the formulation of CSR diagnosis. Diagnostic uncertainty is common in primary care due to the varied clinical presentations and time pressures [44,45]. Moreover, missing serious pathology has been reported by FCPs in primary care [36]. This reaffirms the burden of responsibility on physiotherapists to ensure that sinister or serious pathology is not missed, despite the rarity of such pathology [42]. The burden of responsibility increases the potential for litigation claims among physiotherapists working in primary care FCP roles. The FCP role is unique from other physiotherapy roles, and it generates an additional layer of accountability, emphasising the importance of managing uncertainty and clinical risk [34]. Thus, it is imperative to develop national guidelines to support patients’ and clinicians’ CSR assessment and management decisions across clinical settings.

### Study limitations

Two key study limitations should be raised. Firstly, despite using a robust recruitment strategy, only 63 respondents in the cross-sectional survey of UK physiotherapists participated in the survey. This very low response rate did not meet our sample size calculation and is likely under-representative of the number of UK HCPC physiotherapists assessing CSR in clinical practice. Secondly, no ethnicity data was collected from the survey or interviews, which may limit the representation of our results. Furthermore, it is possible that the interview participants may have unintentionally or intentionally introduced bias into the sample. Self-selection can lead to biased results, as participants with certain attitudes or characteristics may be more likely to volunteer to participate. This bias can have a bidirectional impact, meaning that participants volunteered to participate based on their strong opinions [positive or negative] about the reasons behind CSR assessment decisions.

## Conclusion

Physiotherapists in the UK use large fibre somatosensory testing to establish CSR diagnosis over small fibre somatosensory testing and pain screening questionnaires or tools. The reasons underpinning this decision-making can be grouped into four qualitative themes, perception of role, service constraints, minimising risk, and understanding symptoms. It is important to recognise that large fibre testing is an essential element in establishing CSR diagnosis. However, acknowledging the likely cervical spine nerve root pathomechanisms, physiotherapists should use robust clinical reasoning strategies that incorporate large and small fibre somatosensory testing to establish CSR diagnosis. Furthermore, when diagnosing CSR, physiotherapists should work within their scope of practice and follow their local and regional best practice guidelines. Our mixed methods research study suggests that providing more time to physiotherapists can enhance their assessment practices. However, it may not always be possible to make such changes in healthcare settings like orthopaedic triage centres. Nevertheless, healthcare policies and employers should prioritise offering time-efficient clinical assessment frameworks to support the evidence informed delivery of effective healthcare.

## Supporting information

S1 File Survey design and questions. (DOCX)

S2 File Interview questions and topic guide. (DOCX)

S3 File Fifteen Stages of Thematic Analysis (based on Braun and Clarke, 2014). (DOCX)

S4 File A table presenting the final categories that contributed to each qualitative theme. (DOCX)

S1 Table Good Reporting of A Mixed Methods Study (GRAMMS) checklist. (DOCX)

S2 Table Consolidated criteria for reporting qualitative studies (COREQ): 32-item checklist. (DOCX)
