# Supplementary material for: “You do it to cover your own back”: The assessment of cervical spine radiculopathy among physiotherapists in the United Kingdom: A mixed methods research study
Source: PLoS One. 2025 Jul 2;20(7):e0325922. doi: 10.1371/journal.pone.0325922 (PMC12221016; doi:10.1371/journal.pone.0325922)
Supplement: S1 File — (DOCX) [file pone.0325922.s001.docx]

Supplementary file 1. Survey design and questions

What is this study about?

You are invited to take part in a research study about contemporary assessment strategies used by physiotherapists when reaching a cervical spine radiculopathy diagnosis. Identifying contemporary assessment strategies will shape learning and training development areas for qualified clinicians and future research agendas.

You have been invited to participate in this study because you have accessed it via social media. Please read the Participant Information Sheet (link below) carefully, and ask questions about anything that you don’t understand or want to know more about.

Participation in this research is voluntary. By giving consent to take part in this study you are telling us that you:

- Understand what you have read
- Agree to take part in the research study as outlined below
- Agree to the use of your personal information as described
- You have downloaded a copy of this Participant Information Sheet to keep

**Our Participant Information Sheet can be accessed and downloaded here:** [**https://documentcloud.adobe.com/link/track?uri=urn:aaid:scds:US:28d25181-34a6-47e2-98bd-5f7b3bed68ab**](https://documentcloud.adobe.com/link/track?uri=urn:aaid:scds:US:28d25181-34a6-47e2-98bd-5f7b3bed68ab)

**Online Consent Form**

School of Health Sciences, University of East Anglia.

Study: Understanding cervical spine radiculopathy assessment practices by UK Physiotherapists: An Online Survey.

Name of Lead Researcher: Mr. Michael Mansfield ([michael.mansfield@uea.ac.uk](mailto:michael.mansfield@uea.ac.uk))


**Our Participant Information Sheet can be accessed and downloaded here:**
[**https://documentcloud.adobe.com/link/track?uri=urn:aaid:scds:US:28d25181-34a6-47e2-98bd-5f7b3bed68ab**](https://documentcloud.adobe.com/link/track?uri=urn:aaid:scds:US:28d25181-34a6-47e2-98bd-5f7b3bed68ab)

1.I confirm that I have read and understood the information sheet for the above project. I have had the opportunity to consider the information and asked questions which have been answered to my satisfaction

Yes

No and exit the study

2.I consent voluntarily to be a participant in this project and understand that I can refuse to take part and can withdraw from the project at any time, without having to give a reason

Yes

No and exit the study

3.I consent to the processing of my personal information for the purposes explained to me in the Participation Information Sheet. I understand that such information will be handled in accordance with the terms of the General Data Protection Regulation (GDPR) and the UK Data Protection Act 2018

Yes

No and exit the study

4.I understand that confidentiality and anonymity will be maintained, and it will not be possible to identify me in any research outputs

Yes

No and exit the study

5.I agree to take part in the above study

Yes

No and exit the study

**Professional Identity**

6.Are you a Health Care Profession Council (HCPC) registered physiotherapist practicing in the United Kingdom?

Yes

No

Working practice

7.Please select your professional working area. If you would across multiple areas, please select the working area where you spend most hours

Clinical - Musculoskeletal

Clinical - Orthopaedics

Clinical - Neurology

Clinical - Urgent Care and/or Accident & Emergency

Clinical - Sports

Clinical - Pain management

Clinical - Rheumatology

Clinical - Older adults

None of the above

8.What United Kingdom region do you currently practice in?

England

Northern Ireland

Scotland

Wales

9.Which healthcare sector do you work in (If more than one, please select the sector where you spend most of your working hours)

National Health Service (NHS) - Primary care

National Health Service (NHS) - Secondary care

Charity

Private Practice

Sports

Military

Social enterprise

10.What is your highest educational award?

BSc

Graduate Diploma Physiotherapy

BA

MSc (Pre-registration)

Post Graduate Certificate

Post Graduate Diploma

MSc (Post-registration)

MA

MRes

MPhil

MBA

PhD

Professional Doctorate

11.How many years has it been since you received your highest educational award?

Less than 1 year

1-5 Years

6-10 Years

11-15 Years

16-20 Years

21 Years and above

12.How many years have you worked (as a postgraduate physiotherapist) in a clinical setting assessing and treating people with cervical spine pain with or without radiculopathy?

Less than 1 year

1-5 Years

6-10 Years

11-15 Years

16-20 Years

21 Years and above

13.What proportion of your current working time do you spend assessing and treating people with cervical spine pain with or without radiculopathy?

Less than 10%

10-24%

25-49%

50-74%

75-99%

100%

Diagnosis

14.How confident are you in assessing a person with cervical spine radiculopathy?

Extremely confident

Somewhat confident

Somewhat not confident

Extremely not confident

Diagnostic Methods

15.Do you use these methods when establishing a cervical spine radiculopathy diagnosis?

|  | **Always use** | **Often use** | **Sometimes use** | **Rarely use** | **Heard of this test, but do not use** | **Not heard of this test** |
| --- | --- | --- | --- | --- | --- | --- |
| Pressure Pain Threshold |  |  |  |  |  |  |
| Pressure Detection Threshold |  |  |  |  |  |  |
| Heat Pain Threshold |  |  |  |  |  |  |
| Heat Detection Threshold |  |  |  |  |  |  |
| Cold Pain Threshold |  |  |  |  |  |  |
| Cold Detection Threshold |  |  |  |  |  |  |
| Temporal Summation |  |  |  |  |  |  |
| Conditioned Pain Modulation |  |  |  |  |  |  |
| Vibration Detection Threshold |  |  |  |  |  |  |
| Mechanical Pain Sensitivity |  |  |  |  |  |  |
| Dynamic Mechanical Allodynia |  |  |  |  |  |  |
| Reflexes |  |  |  |  |  |  |
| Myotomes Testing or Manual Muscle Testing |  |  |  |  |  |  |
| Spurling’s Test Manoeuvre |  |  |  |  |  |  |
| “Neural dynamic test”  *Examples may include: Upper Limb Tension Test (ULTT)* *Upper Limb Neural Test (ULNT))* |  |  |  |  |  |  |
| Light touch test (light touch sensation) |  |  |  |  |  |  |

16.Is there another **physical assessment method** which you have heard of or use when establishing a cervical spine radiculopathy diagnosis which is not listed in the previous question?

*(Enter "No" if there is not another physical assessment method)*

**Possible Barriers, Somatosensory Assessment Methods**

17.Why do you think the somatosensory assessment methods listed below are *rarely used or not used* in clinical practice when establishing a cervical spine radiculopathy diagnosis?

***(Pressure Pain Threshold; Pressure Detection Threshold; Heat Pain Threshold; Heat Detection Threshold; Cold Pain Threshold; Cold Detection Threshold; Temporal Summation; Conditioned Pain Modulation; Vibration Detection Threshold; Mechanical Pain Sensitivity; Dynamic Mechanical Allodynia)***


Select all which apply

Time to collect this data

Cost of equipment

Reduced confidence using the equipment or interpreting data collected

They are more useful for researchers than clinicians in practice

Prioritising other assessment metrics when forming a cervical spine radiculopathy diagnosis

Availability of equipment

**Possible Facilitators, Somatosensory Assessment Methods**

18.What do you think could improve the use of somatosensory assessment methods listed below when establishing a cervical spine radiculopathy diagnosis?

***(Pressure Pain Threshold; Pressure Detection Threshold; Heat Pain Threshold; Heat Detection Threshold; Cold Pain Threshold; Cold Detection Threshold; Temporal Summation; Conditioned Pain Modulation; Vibration Detection Threshold; Mechanical Pain Sensitivity; Dynamic Mechanical Allodynia)***

Select all which apply

Additional time in clinic to complete

Further training on how to use and data interpretation

Further training on when to use

Reduction in equipment costs

Diagnosis Methods - Imaging

19.Do you use these imaging modalities when establishing a cervical spine radiculopathy diagnosis?

|  | **Always use** | **Often use** | **Sometimes use** | **Rarely use** | **Heard of this imaging modality, but do not use** | **Not heard of this imaging modality** |
| --- | --- | --- | --- | --- | --- | --- |
| Magnetic Resonance Imaging (MRI) |  |  |  |  |  |  |
| Computed Tomography (CT) |  |  |  |  |  |  |
| Nerve Conduction Studies (NCS) |  |  |  |  |  |  |
| Plain Radiograph |  |  |  |  |  |  |

**Diagnosis, Screening Tools or Questionnaires**

20.Do you use these screening tools or questionnaires when making a diagnosis of cervical spine radiculopathy?

|  | **Always use** | **Often use** | **Sometimes use** | **Rarely use** | **Heard of this but do not use** | **Not heard of this** |
| --- | --- | --- | --- | --- | --- | --- |
| Douleure Neuropathique en 4 questions (DN4) |  |  |  |  |  |  |
| The Leeds Assessment of  Neuropathic Symptoms and Signs (LANSS) |  |  |  |  |  |  |
| PainDETECT |  |  |  |  |  |  |
| Neuropathic Pain Questionnaire (NPQ) |  |  |  |  |  |  |
| ID-Pain |  |  |  |  |  |  |

21.Why do you think these screening tools or questionnaires are *rarely used or not used* in clinical practice when establishing a cervical spine radiculopathy diagnosis

Select all which apply

Reduced time available to collect this data

Confidence using or interpreting data collected in screening tools

They are more useful for researchers than clinicians in practice

Prioritising other assessment metrics when forming a cervical spine radiculopathy diagnosis

**Possible Facilitators, Screening Tools or Questionnaires**

22.What do you think could improve the usage of **screening tools or questionnaires** when establishing a cervical spine radiculopathy diagnosis?

Select all which apply

Free online access to screening tools

Additional time in clinic to complete

Further training on how to use and data interpretation

Further training on when to use

**Completion and Exit Page**

Thank you for taking the time to complete this survey.

As the data is anonymised at the point of submission of the survey, you will not be able to withdraw your responses (data) from the study after that point once you submit your survey.
 **If you have any queries or concerns about this survey, please contact Michael Mansfield (**[**Michael.mansfield@uea.ac.uk**](mailto:Michael.mansfield@uea.ac.uk)**)**

Please ensure you have clicked the submit button to exit the survey

23.A follow up interview study will be conducted to explore the responses by participants from this survey.

Please provide your email address below if you are happy to be contacted about this interview study:

24.If you are happy to receive a report of the survey results, please enter your email address below:
