# Supplementary material for: “You do it to cover your own back”: The assessment of cervical spine radiculopathy among physiotherapists in the United Kingdom: A mixed methods research study
Source: PLoS One. 2025 Jul 2;20(7):e0325922. doi: 10.1371/journal.pone.0325922 (PMC12221016; doi:10.1371/journal.pone.0325922)
Supplement: S2 File — (DOCX) [file pone.0325922.s002.docx]

**Supplementary file 2. Interview questions and topic guide**

| **Key quantitative finding from survey** | **Questions** |
| --- | --- |
| N/A | *WARM UP STATEMENTS AND QUESTIONS:*  Thanking the participant  Outlining that there were no right or wrong answers to any of the questions  Only interested in their thoughts, experiences and opinions  Tell me about your current working clinical environment working as a Physiotherapist.  What presentations do you typically see?  What age groups do predominantly work with?  _________________________________________  What is your experience of assessing and/or treating people with cervical spine radiculopathy?  Prompt: How often do you assess and/or treat people with cervical spine radiculopathy? |
| Nearly all UK physiotherapists who completed the survey indicated they always use myotomes (muscle strength), reflexes and light touch sensation assessment strategies when establishing a CSR diagnosis | Can you tell me about the assessment strategies you use to diagnose cervical spine radiculopathy (CSR)?   - Are there particular clinical signs, symptoms or features that outweigh others in establishing a CSR diagnosis? - **Why are these important?** - Are there any instances when you might adapt this assessment practice? |
| Pain screening questionnaires are not routinely used in practice when establishing CSR | What is your approach when assessing a person’s CSR sensory experience?   - What prompts you to complete this? - Have you always done this?   Why is that important in forming a CSR diagnosis?  Stepping away from your own practice, our survey suggests that UK Physiotherapists do not use pain screening questionnaires when establishing a cervical spine radiculopathy diagnosis. We’d like you to help us unpack why this may be the case. |
| Reasons why physiotherapists do not use pain screening questionnaires when establishing CSR:  *Time in the clinic; Questionnaire availability;*  *Knowledge around interpreting pain screening questionnaire findings* | Our survey explored the reasons why UK Physiotherapists do not use pain screening questionnaires in clinical practice  Again, we’d like you to help us unpack the reasons why physiotherapists do not use pain screening questionnaires. What are your thoughts about this finding?  Does this relate to your clinical practice?   - Why? - Tell me more about this |
| Reasons why physiotherapists do not use somatosensory tests when establishing CSR:  *Time in the clinic; Equipment availability;*  *Knowledge around interpreting somatosensory test parameter findings; Usefulness in clinical setting* | What are your thoughts on the reasons why physiotherapists do not use these test parameters?  Does this relate to your clinical practice?   - **Why?** - Tell me more about this |
| **Facilitators** | |
| Reasons which might support the use of pain screening tools in clinic:  *Free online access; additional time in clinic; further training on how to use and data interpretation* | Our survey explored the reasons why UK Physiotherapists may adopt pain screening questionnaires when establishing CSR  What are your thoughts on what could facilitate the use of these pain screening questionnaires in practice?  How could these strategies be implemented in clinical practice? |
| Reasons which might support the use of somatosensory test parameters in clinic:  *Free online access; additional time in clinic; further training on how to use and data interpretation* | Our survey explored what might facilitate UK Physiotherapists to adopt somatosensory test parameters when establishing CSR  What are your thoughts on might facilitate the use of these somatosensory test parameters in practice?  How could these strategies be implemented in clinical practice? |
