# Supplementary material for: “You do it to cover your own back”: The assessment of cervical spine radiculopathy among physiotherapists in the United Kingdom: A mixed methods research study
Source: PLoS One. 2025 Jul 2;20(7):e0325922. doi: 10.1371/journal.pone.0325922 (PMC12221016; doi:10.1371/journal.pone.0325922)
Supplement: S3 File — (DOCX) [file pone.0325922.s003.docx]

Supplementary file 3. Fifteen Stages of Thematic Analysis (based on Braun and Clarke, 2014)

| **Stage** | **Activity** | **Criteria** |
| --- | --- | --- |
|  | Transcription | The data have been transcribed to an appropriate level of detail, and the transcripts have been checked against the tapes for ‘accuracy’ |
| 2-6. | Coding | Each data item has been given equal attention in the coding process  Themes have not been generated from a few vivid examples (an anecdotal approach), but instead the coding process has been thorough, inclusive and comprehensive  All relevant extracts for all each theme have been collated  Themes have been checked against each other and back to the original data set  Themes are internally coherent, consistent, and distinctive |
| 7-10. | Analysis | Data have been analysed – _interpreted, made sense of - rather than just paraphrased or described  Analysis and data match each other – _the extracts illustrate the analytic claims  Analysis tells a convincing and well-organised story about the data and topic  A good balance between analytic narrative and illustrative extracts is provided |
| 11. | Overall | Enough time has been allocated to complete all phases of the analysis adequately, without rushing a phase or giving it a once-over-lightly |
| 12-15. | Written report | The assumptions about, and specific approach to, thematic analysis are clearly explicated  There is a good fit between what you claim you do, and what you show you have done – _i.e., described method and reported analysis are consistent  The language and concepts used in the report are consistent with the epistemological position of the analysis  The researcher is positioned as active in the research process |
