# Supplementary material for: “You do it to cover your own back”: The assessment of cervical spine radiculopathy among physiotherapists in the United Kingdom: A mixed methods research study
Source: PLoS One. 2025 Jul 2;20(7):e0325922. doi: 10.1371/journal.pone.0325922 (PMC12221016; doi:10.1371/journal.pone.0325922)
Supplement: S4 File — (DOCX) [file pone.0325922.s004.docx]

Supplementary file 4. A table presenting the final categories that contributed to each qualitative theme.

| **Main theme** | **Contributing categories** |
| --- | --- |
| **Perception of role** | Convince me  Knowledge and education  Clinical experience  Expectations |
| **Service constraints** | Time  Software  Public – Private Sector  Equipment |
| **Minimise risk** | External influencers: Medico-legal and healthcare managers  Internal factors |
| **Understand symptoms** | Pain  Nerve descriptors |
