# Supplementary material for: “You do it to cover your own back”: The assessment of cervical spine radiculopathy among physiotherapists in the United Kingdom: A mixed methods research study
Source: PLoS One. 2025 Jul 2;20(7):e0325922. doi: 10.1371/journal.pone.0325922 (PMC12221016; doi:10.1371/journal.pone.0325922)
Supplement: S1 Table — (DOCX) [file pone.0325922.s005.docx]

**Supplementary Table 1. Good Reporting of A Mixed Methods Study (GRAMMS) checklist**

| **Guideline** | **Pages** |
| --- | --- |
| Describe the justification for using a mixed methods approach to the research question | **5-6** |
| Describe the design in terms of the purpose, priority and sequence of methods | **5-6** |
| Describe each method in terms of sampling, data collection and analysis | **6-11** |
| Describe where integration has occurred, how it has occurred and who has participated in it | **11** |
| Describe any limitation of one method associated with the present of the other method | **34** |
| Describe any insights gained from mixing or integrating methods | **11** |
